# Non-Hermitian fractional quantum Hall states

**DOI:** 10.1038/s41598-019-53253-8

**Published:** 2019-11-15

**Authors:** Tsuneya Yoshida, Koji Kudo, Yasuhiro Hatsugai

**Affiliations:** 1 Graduate School of Pure and Applied Sciences, University of Tsukuba, Tsukuba, Ibaraki, 305-8571 Japan; 2 Department of Physics, University of Tsukuba, Tsukuba, Ibaraki, 305-8571 Japan

**Keywords:** Quantum Hall, Topological insulators

## Abstract

We demonstrate the emergence of a topological ordered phase for non-Hermitian systems. Specifically, we elucidate that systems with non-Hermitian two-body interactions show a fractional quantum Hall (FQH) state. The non-Hermitian Hamiltonian is considered to be relevant to cold atoms with dissipation. We conclude the emergence of the non-Hermitian FQH state by the presence of the topological degeneracy and by the many-body Chern number for the ground state multiplet showing *C*_tot_ = 1. The robust topological degeneracy against non-Hermiticity arises from the manybody translational symmetry. Furthermore, we discover that the FQH state emerges without any repulsive interactions, which is attributed to a phenomenon reminiscent of the continuous quantum Zeno effect.

## Introduction

In these decades, a variety of novel phenomena have been discovered which arise from topological properties in the bulk^1–13^. In particular, the topological ordered phases^14^ exhibit striking topological phenomena because of correlation effects and the topological properties. One of the representative examples of topological ordered phases is a fractional quantum Hall (FQH) phase^15–17^ or a fractional Chern insulator^18–23^ which hosts anyons obeying fractional statistics due to the topological degeneracy of the ground states. The platforms of topological ordered phases extend to bosonic or spin systems. The toric codes for two-^24,25^ and three-dimensional systems^26^ exemplify the emergence of topological ordered phase in spin systems whose relevance of correlated compounds has been discussed recently^27,28^. These topological ordered phases also attract much attention in terms of application to the quantum computations.

Along with the above progress, recent development of technology has pioneered a new type of topological systems, non-Hermitian topological systems. Extensive studies in these years have discovered various intriguing phenomena described by topological properties of quadratic non-Hermitian matrices. For instance, it has been elucidated that non-Hermiticity may induce a topological phase which does not have its Hermitian counterpart^29–32^. Furthermore, non-Hermiticity induces novel gapless excitations in the bulk (e.g., exceptional points^33–39^, symmetry-protected exceptional rings^40–46^ etc.) which arise from the defectiveness of the Hamiltonian. In addition, non-Hermiticity may induce a unique bulk-boundary correspondence^47–52^ due to the non-Hermitian skin effect.

The above two progresses pose the following crucial question: *what are impacts of non-Hermiticity on topologically ordered phases?* In particular, it is considered to be significant to elucidate the fate of the topological degeneracy which is source of anyons for Hermitian cases. In spite of such significant open questions, there are few works addressing non-Hermitian topological ordered phases.

In this paper, we address the above issue, providing a new direction in the study of non-Hermitian topological phases. Specifically, we demonstrate the emergence of non-Hermitian FQH states in a two-dimensional system with non-Hermitian interactions where spinless fermions are coupled to Abelian gauge fields. This system is considered to be relevant to cold atoms with two-body loss. We conclude the emergence of non-Hermitian FQH states by combining the following two results: direct computation of the Chern number *C*_tot_ indicates that the ground state multiplet is characterized with *C*_tot_ = 1; the topological degeneracy is robust against non-Hermitian interactions, which arises from many-body translational symmetry. Furthermore, we discover a novel phenomenon for which non-Hermiticity is essential; the FQH state emerges without the repulsive interactions. We find that this intriguing phenomenon arises from interplay between the kinetic term and the dissipative interactions which is reminiscent of the continuous quantum Zeno effect. This unconventional mechanism of the bulk gap may provide a new way to access exotic topological ordered phases.

## Setup and Method

The non-Hermitian Hamiltonian analyzed in this paper is shown in Eq. (3) which is considered to be relevant to cold atoms; the inelastic scattering results in the two-body interactions with the prefactor $V\in {\mathbb{C}}$ [see Eq. (3c)]. In order to see the details, we start with spinless fermions in Abelian gauge potentials which are decoupled with the environment. After that we take into account the coupling with the environment, yielding a non-Hermitian Hamiltonian.

Firstly, we note that the following square lattice system with Abelian gauge fields may be realized for cold atoms

1 $${H}_{0}=-\sum _{\langle i,j\rangle }\,{t}_{0}{e}^{i{\varphi }_{ij}}{c}_{i}^{\dagger }{c}_{j}+{V}_{R}\sum _{\langle i,j\rangle }\,{n}_{i}{n}_{j}\mathrm{.}$$

**Figure 1:**
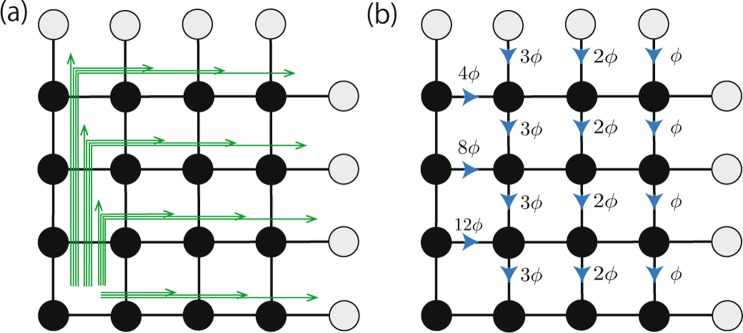
Sketch of the model and the string gauge for *N*_*x*_ = *N*_*y*_ = 4. We impose the periodic boundary condition for *x*- and *y*-direction. The green arrows in panel (a) represent strings specifying the Peierls phase *ϕ*_*ij*_ = 2*πϕn*_*ij*_. Here, *n*_*ij*_ denotes the number of string penetrating the bond connecting sites *i* and *j*, and *ϕ* denotes the flux density *ϕ* = *N*_*ϕ*_/*N*_*x*_*N*_*y*_. Panel (b) illustrates the corresponding Peierls phase; when the fermion hops along the blue arrow, it acquires the phase factor *ϕ*_*ij*_. When *ϕ* is multiple of *N*_*x*_^−1^, the string gauge is reduced to the Landau gauge.

Here, ${c}_{i}^{\dagger }$ creates a spinless fermion at site *i* = (*i*_*x*_,*i*_*y*_) of the two-dimensional system. If necessary, we rewrite ${c}_{i}^{\dagger }$ as ${c}_{{i}_{x}{i}_{y}}^{\dagger }$. *t*_0_ denotes the hopping between sites. The phase factor *ϕ*_*ij*_ describes the flux penetrating the plaquet. In this paper, we employ the string gauge^53^ (see Fig. 1). We define the flux density as *ϕ* $:\,=$ *N*_*ϕ*_/*N*_*x*_*N*_*y*_ where *N*_*ϕ*_ denotes the number of flux quanta penetrating the *N*_*x*_ × *N*_*y*_-square lattice. The filling factor is defined as *ν* $:\,=$ *Nf*/*N*_*ϕ*_ where *N*_*f*_ denotes the number of fermions. For fabrication of the above system with cold atoms, the following two ingredients are essential: nontrivial hopping inducing Landau bands and the repulsive interactions. The former ones are introduced by rotating the system^54–58^ or by optically synthesized gauge fields^59^. The repulsive interaction (*V*_*R*_ > 0) may be fabricated by a Feshbach resonance^60,61^.

Now, let us take into account the coupling with the environment induced by inelastic scattering^62–68^. The time-evolution of such an open quantum system is governed by the Lindblad equation:

2 $${\partial }_{t}\rho (t)=-\,i[{H}_{0},\rho (t)]-\frac{1}{2}\gamma \sum _{k}\,({L}_{k}^{\dagger }{L}_{k}\rho (t)+\rho (t){L}_{k}^{\dagger }{L}_{k}-2{L}_{k}\rho {L}_{k}^{\dagger }),$$

where *ρ*(*t*) denotes the density matrix of the system. *L*_*k*_’s are Lindblad operators describing the loss with the rate *γ* > 0. Specifically, we set *L*_*k*_ → ${c}_{i}{c}_{i+{{\boldsymbol{e}}}_{x}}$, ${c}_{i}{c}_{i+{{\boldsymbol{e}}}_{y}}$ because the Feshbach resonance induces the two-particle loss^62–68^. Here ***e***_*x*(*y*)_ denotes the unit vector for each direction, and the lattice constant is set to unity. When we focus on the short-time evolution, the last term describing the quantum-jump is negligible^66–69^. In this case, we can see that the time-evolution is described by

3a $${\partial }_{t}\rho (t)=-\,i({H}_{{\rm{eff}}}\rho (t)-\rho (t){H}_{{\rm{eff}}}^{\dagger }),$$

3b $${H}_{{\rm{eff}}}={H}_{{\rm{kin}}}+{H}_{{\rm{int}}},$$

with

3c $${H}_{{\rm{kin}}}=-\,\sum _{\langle i,j\rangle }\,{t}_{0}{e}^{i{\varphi }_{ij}}{c}_{i}^{\dagger }{c}_{j},\,{H}_{{\rm{int}}}=V\,\sum _{\langle i,j\rangle }\,{n}_{i}{n}_{j}\mathrm{.}$$

We note that the interaction strength takes a complex value; $V={V}_{R}-i\frac{\gamma }{2}$ with *V*_*R*_ ≥ 0, which makes the Hamiltonian non-Hermitian ${H}_{{\rm{eff}}}\ne {H}_{{\rm{eff}}}^{\dagger }$. The short-time evolution can be elucidated by diagonalizing the *H*_eff_ defined in Eq. (3b).

Because treating the large size system is numerically difficult, we simplify the Hamiltonian (3b) with calculating the pseudo-potential^70^. With this approximation, the Hamiltonian is simplified as

4a $${H^{\prime} }_{{\rm{eff}}}=-\,\sum _{\langle i,j\rangle }\,{t}_{0}{e}^{i{\varphi }_{ij}}{\tilde{c}}_{i}^{\dagger }{\tilde{c}}_{j}+V\,\sum _{\langle i,j\rangle }\,{\tilde{c}}_{i}^{\dagger }{\tilde{c}}_{j}^{\dagger }{\tilde{c}}_{j}{\tilde{c}}_{i},$$

with

4b $${H}_{{\rm{kin}}}|{\varphi }_{\alpha }\rangle =|{\varphi }_{\alpha }\rangle {\varepsilon }_{\alpha },\,{\tilde{c}}_{i}^{\dagger }=\sum _{\alpha }\,^{\prime} {\varphi }_{i\alpha }^{\ast }{d}_{\alpha }^{\dagger }\mathrm{.}$$

Here, |*ϕ*_*α*_〉 denotes the eigenstate of *H*_kin_ ($|{\varphi }_{\alpha }\rangle :\,={\sum }_{j}\,{\varphi }_{j\alpha }{c}_{j}^{\dagger }\mathrm{|0}\rangle$). We label the eigenvalues ${\varepsilon }_{\alpha }$ so that the relation ${\varepsilon }_{1}$  ≤  ${\varepsilon }_{2}$  ≤ $\cdots$ is satisfied. ${d}_{\alpha }^{\dagger }$ creates the fermion of the eigenstate *α*. ∑′_*α*_ denotes the summation over states satisfying ${\varepsilon }_{\alpha }$ ≤ $\varepsilon$*N*_keep_; e.g., for *N*_keep_ = *N*_*ϕ*_ [*N*_keep_ = 2*N*_*ϕ*_], the summation is taken over the lowest Landau levels (LLs) [the lowest and the second lowest LLs], respectively. For *N*_keep_ = 2*N*_*ϕ*_, we can take into account effects of Landau band mixing which induces an intriguing phenomena as we see below.

For the numerical computation, we set parameters as |*V*| = *t*_0_ = 1, *ν* = 1/3, and *N*_*x*_ = *N*_*y*_ = *N*.

## Results

### Hermitian case

Here we briefly review the results of the Hermitian system (Im*V* = 0) for *ν* = 1/3 where FQH states have been observed^16,18–23,71–74^. In this case, three-fold degeneracy is observed for the ground state multiplet which is separated by the bulk gap. Computing the Chern number *C*_tot_ for the ground state multiplet yields *C*_tot_ = 1, which characterizes the topology of the FQH state with the Hall conductance *σ*_*xy*_ = 1/3. For more details, see Sec. I of Supplemental Material.

### Non-Hermitian case

Now we introduce the imaginary-part Im*V* < 0, which makes the system non-Hermitian. Let us start with the definitions of the ground states and the energy gap because the energy spectrum of the non-Hermitian Hamiltonian becomes complex. We define the ground states with the minimum value of the real-part^66^. For our system, these states also have the longest lifetime *τ* (~−1/Im*E* with Im*E* < 0). Correspondingly, the energy gap is defined as Δ = Re*E*_*e*_ − Re*E*_*g*_ which is natural extension of the Hermitian case. Here, *E*_*g*_ (*E*_*e*_) denotes the energy eigenvalue of the ground state (the first excited state), respectively. In the following, we numerically show that the FQH state survives even under non-Hermiticity by setting *V* = exp(−*in*_*θ*_*π*/10) with *n*_*θ*_ = 0, …, 5.

**Figure 2:**
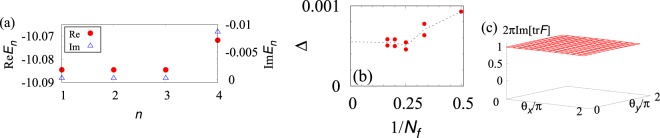
Numerical results for *n*_*θ*_ = 2 with *V* = exp(−*in*_*θ*_*π*/10). (**a**) The real- and the imaginary-part of the energy eigenvalues. The data are obtained for *n*_*θ*_ = 2, *N*_*ϕ*_ = *N* = 9, and *N*_keep_ = 2*N*_*ϕ*_. (**b**) The bulk gap as a function of *N*_*f*_. In this plot, the size of the system is chosen so that the flux density *ϕ* = *N*_*ϕ*_/*N*^2^ satisfies 1/45 ≤ *ϕ* ≤ 1/40. The data of panel (b) are obtained for *N*_keep_ = *N*_*ϕ*_. However, the difference from the gap for *N*_keep_ = 2*N*_*ϕ*_ is less than $\delta \Delta \lesssim {10}^{-5}{t}_{0}$. (**c**) The imaginary-part of the Berry curvature tr*F* as a function of *θ*_*x*_ and *θ*_*y*_ for *N*_keep_ = *N*_*ϕ*_. For the computation, we divide the two-dimensional space of *θ*’s into *N*_*θ*_ × *N*_*θ*_-mesh with *N*_*θ*_ = 14.

As a first step, we focus on the case for *n*_*θ*_ = 2. In Fig. 2(b), we plot the energy spectrum *E*_*n*_ where *n* labels the states such that Re*E*_1_ ≤ Re*E*_2_ ≤ $\cdots$ holds. Figure 2(a) indicates that the three-fold degeneracy can be observed even in the presence of non-Hermitian term. The robustness is attributed to many-body translational symmetry, which we discuss below. Besides that, in this figure, we can confirm that the lifetime of the ground states is longer than that of excited states. We note that the energy gap observed in this figure remains finite in the thermodynamic limit, which can be seen in Fig. 2(b).

From the above numerical results of the bulk gap and the topological degeneracy, one can expect that the FQH phase survives even in the presence of the non-Hermitian term. To confirm this, we address the characterization of the topology of the ground states by computing the many-body Chern number for the non-Hermitian case which is defined as follows:

5a $${C}_{{\rm{tot}}}=\int \frac{d{\theta }_{x}d{\theta }_{y}}{2\pi i}{\rm{tr}}F({\theta }_{x},{\theta }_{y}),$$

5b $${F}_{nm}({\theta }_{x},{\theta }_{y})={\epsilon }_{\mu \nu L}{\langle {\partial }_{\mu }{\varPsi }_{n}|{\partial }_{\nu }{\Psi }_{m}\rangle }_{R}\mathrm{.}$$

Here, we have imposed the twisted boundary condition: ${c}_{{N}_{x}+\mathrm{1,}{i}_{y}}^{\dagger }={e}^{i{\theta }_{x}}{c}_{\mathrm{1,}{i}_{y}}^{\dagger }$ and ${c}_{{i}_{x},{N}_{y}+1}^{\dagger }={e}^{i{\theta }_{y}}{c}_{{i}_{x}\mathrm{,1}}^{\dagger }$. The integral is taken over 0 ≤ *θ*_*x*(*y*)_ < 2*π*, respectively. *F*(*θ*_*x*_,*θ*_*y*_) denotes the Berry curvature defined by twisting the boundary conditions. ∂_*μ*_ $:\,=$ ∂/∂*θ*_*μ*_. ${\varepsilon }_{\mu \nu }$ (*μ*, *ν* = *x*, *y*) is an anti-symmetric matrix with ${\varepsilon }_{xy}$ = 1. |Ψ_*n*_〉_*R*_ and _*L*_〈Ψ_*n*_| denote ground states with *n* = 1, 2, 3. The former (latter) ones are right (left) eigenvectors. The summation is taken over repeated indices. We note that the Chern number defined above takes integer (for the proof, see Sec. II of Supplemental Material). This fact indicates that the only imaginary-part of the Berry curvature contributes to the Chern number *C*_tot_; taking the integral, the contribution from the real-part of the Berry curvature generically vanishes. Employing the method introduced in refs ^75^ and^76^, we obtain Im[tr*F*]. In Fig. 2(c), we can see that the integrand Im[tr*F*]/2*π* becomes almost constant. Evaluating the integration, we obtain *C*_tot_ = 1.

The above data of the bulk gap, the ground state degeneracy, and the Chern number suggest that the ground state is topologically identical to the FQH state with *σ*_*xy*_ = 1/3 for the Hermitian case.

**Figure 3:**
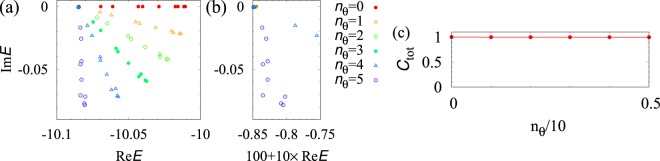
(**a**) Energy spectrum for several values of *n*_*θ*_ defining interaction with *V* = exp(−*in*_*θ*_*π*/10). Panel (b) shows the magnified data. (**c**) Chern number as a function of *n*_*θ*_. The data are obtained for *N* = 9 and *N*_keep_ = 18 where both of the lowest and the second lowest LLs are taken into account. We note that for *n*_*θ*_ = 5 the interaction strength *V* becomes pure imaginary *V* = −*i*.

In a similar way, we can analyze the system for the other cases of interaction strength. The results are summarized in Fig. 3. This figure indicates that the FQH state observed for *n*_*θ*_ = 2 is adiabatically connected to the one for the Hermitian case; Fig. 3(a,b) show that the bulk gap remains finite with decreasing *n*_*θ*_; Fig. 3(c) indicates that the topological properties do not change.

Intriguingly, Fig. 3(b) indicates that the bulk gap opens even for Re*V* = 0, implying the potential presence of the FQH state without the repulsive interaction. The details of this issue are addressed below.

### Robustness of the ground state degeneracy against non-Hermitian interactions

So far, we have numerically observed the three-fold degeneracy of the ground states even in the presence of the non-Hermitian term [see Figs 2(a) and 3(a)]. This three-fold degeneracy is due to many-body translational symmetry. Namely, the degeneracy multiple of *ν*^−1^ = 2*m* + 1 ($m\in {\bf{Z}}$) is observed for arbitrary many-body interaction preserving the translational symmetry. In the following, we discuss the details.

To see this we focus on the case where *N*_*x*_ = *N*_*y*_ and *ϕ* = *n*_*x*_/*N*_*x*_ (${n}_{x}\in {\bf{Z}}$) holds. Due to the latter condition, the string gauge is reduced to the Landau gauge. In this case, the kinetic term *H*_kin_ preserves the translation symmetry along the *y*-axis. Namely, the following condition holds; *T*_*y*_*H*_kin_*T*_*y*_^−1^ = *H*_kin_ where *T*_*y*_ is the translation operator satisfying ${T}_{y}{c}_{{i}_{x}{i}_{y}}^{\dagger }{T}_{y}^{-1}={c}_{{i}_{x}{i}_{y}+1}^{\dagger }$.

Because of the translation symmetry of *H*_kin_, one may take the simultaneous eigenstates of *H*_kin_ and *T*_*y*_;

6a $${H}_{{\rm{kin}}}|{\phi }_{\alpha }({k}_{y})\rangle ={\varepsilon }_{\alpha }|{\phi }_{\alpha }({k}_{y})\rangle ,$$

6b $${T}_{y}|{\phi }_{\alpha }({k}_{y})\rangle ={e}^{-i{k}_{y}}|{\phi }_{\alpha }({k}_{y})\rangle ,$$

where *k*_*y*_ denotes the momentum along the *y*-axis (0 ≤ *k*_*y*_ < 2*π*). Here, let us consider the following gauge transformation: ${U}_{G}{c}_{{j}_{x}{j}_{y}}^{\dagger }{U}_{G}^{\dagger }={e}^{-i2\pi \varphi {j}_{y}}{c}_{{j}_{x}{j}_{y}}^{\dagger }$, where *U*_*G*_ is an unitary operator. Applying the gauge transformation to the eigenstate |*φ*_*α*_(*k*_*y*_)〉, we obtain

7 $${T}_{y}{U}_{G}|{\phi }_{\alpha }({k}_{y})\rangle ={e}^{-i({k}_{y}-2\pi \varphi )}{U}_{G}|{\phi }_{\alpha }({k}_{y})\rangle ,$$

which means that applying *U*_*G*_ shifts the momentum by Δ*k*_*y*_ := −2*πϕ*. For the derivation of Eq. (7), see Sec. III of Supplemental Material.

Equation (7) elucidates that the many-body translational symmetry results in the degeneracy multiple of *ν*^−1^. This can be seen by noticing the following relation

8 $$\begin{array}{c}\langle {\phi }_{{\alpha }_{1}}({k}_{y1}),\ldots ,{\phi }_{{\alpha }_{{N}_{f}}}({k}_{y{N}_{f}})|{H}_{{\rm{int}}}|{\phi }_{{\beta }_{1}}({k^{\prime} }_{y1}),\ldots ,{\phi }_{{\beta }_{{N}_{f}}}({k^{\prime} }_{y{N}_{f}})\rangle \\ \,=\,\langle {\phi }_{{\alpha }_{1}}({k}_{y1}),\ldots ,{\phi }_{{\alpha }_{{N}_{f}}}({k}_{y{N}_{f}})|{U}_{G}^{\dagger }{H}_{{\rm{int}}}{U}_{G}|{\phi }_{{\beta }_{1}}({k^{\prime} }_{y1}),\ldots ,{\phi }_{{\beta }_{{N}_{f}}}({k^{\prime} }_{y{N}_{f}})\rangle \\ \,=\,\langle {\phi }_{{\alpha }_{1}}({k}_{y1}+\Delta {k}_{y}),\ldots ,{\phi }_{{\alpha }_{{N}_{f}}}({k}_{y{N}_{f}}+\Delta {k}_{y})|{H}_{{\rm{int}}}|{\phi }_{{\beta }_{1}}({k^{\prime} }_{y1}+\Delta {k}_{y}),\ldots ,{\phi }_{{\beta }_{{N}_{f}}}({k^{\prime} }_{y{N}_{f}}+\Delta {k}_{y})\rangle ,\end{array}$$

which indicates that the matrix element for the subspace labeled by the total momentum $K={\sum }_{l}\,{k}_{yl}$ equals to the one for the subspace labeled by *K*′ = *K* + Δ*k*_*y*_*N*_*f*_. Because the shift of the momentum is rewritten as Δ*k*_*y*_*N*_*f*_ = −2*πϕN*_*f*_ = −2*πν*, we can see that the degeneracy of each eigenvalue is multiple of *ν*^−1^.

**Figure 4:**
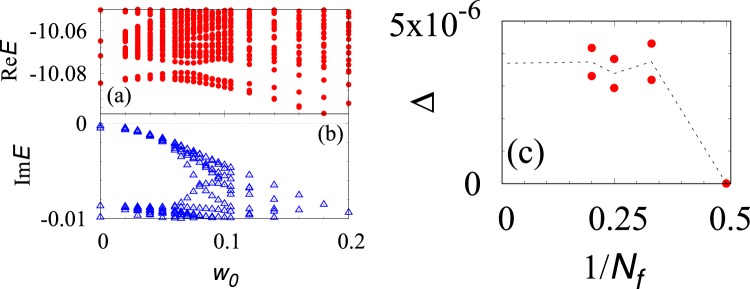
(**a**,**b**) The real- [imaginary-] part of the energy eigenvalues as functions of disorder strength. Turning on disorder *w*_0_ splits the three-fold degeneracy observed for *w*_0_. The data are obtained for *N*_*ϕ*_ = *N* = 9 and *n*_*θ*_ = 2 with *V* = exp(−*in*_*θ*_*π*/10). (**c**) The bulk gap as a function of *N*_*f*_ which is obtained for *H*_ptb_ [see Eq. (10)]. Energy difference of the ground state multiplet is of the order of 10^−13^ *t*_0_ which is much smaller than the energy gap.

In the above we have seen the relation between the topological degeneracy and the many-body translational symmetry. In order to support this numerically, we demonstrate that breaking the translational symmetry splits the degeneracy. Specifically, we compute the energy spectrum in the presence of the following disorder

9 $${H}_{{\rm{dis}}}=\sum _{i}\,{w}_{i}{\tilde{c}}_{i}^{\dagger }{\tilde{c}}_{i},$$

where *w*_*i*_ takes a random value satisfying −*w*_0_/2 ≤ *w*_*i*_ ≤ *w*_0_/2 at each site. In Fig. 4(a) [(b)], the real- [imaginary-] part of the energy eigenvalues are plotted against disorder strength *w*_0_, respectively. These figures indicate that breaking the translational symmetry lifts the three-fold degeneracy of the ground states.

The above results indicate that the many-body translational symmetry results in the robustness of topological degeneracy against non-Hermiticity. Our numerical data elucidate that the topological degeneracy can be observed for 1/45 ≤ *ϕ* < 1/40 where the string gauge cannot be reduced to the Landau gauge.

### FQH state without the repulsive interaction

Figure 3(b,c) imply that the FQH state emerge without the repulsive interaction. In the following, we elucidate the origin of the FQH state for Re*V* = 0.

Firstly, we point out that the origin of the gap is the interplay between the kinetic term *H*_kin_ and the non-Hermitian interaction *H*_int_ (i.e., the mixing between Landau bands). Applying the perturbation theory, we obtain the following Hamiltonian acting on the space spanned by the states in the lowest LLs,

10 $${H}_{{\rm{ptb}}}={P}_{0}{H}_{{\rm{int}}}{P}_{0}+{P}_{0}{H}_{{\rm{int}}}{P}_{1}\frac{1}{{E}_{g}^{0}-{H}_{{\rm{kin}}}}{P}_{1}{H}_{{\rm{int}}}{P}_{0}\mathrm{.}$$

Here, *P*_*n*_ denotes the projection operator to the subspace where *n*-fermions are excited to the second lowest LLs. *E*_*g*_^0^ is the ground state energy for *V* = 0. We have omitted the constant term arising from *P*_0_*H*_kin_*P*_0_. Noticing that the prefactor of the last term is (Im*V*)^2^/*ħω*_0_ > 0, we can see that the last term serves as the repulsive interaction for Re*V* = 0. Here *ħω*_0_ denotes the energy gap between the lowest LLs and the second lowest LLs for *V* = 0.

Diagonalizing the effective Hamiltonian (10), we plot the energy gap Δ as a function of *N*_*f*_ in Fig. 4(c). This figure indicates that the energy gap remains finite in the thermodynamic limit. We also note that the three-fold degeneracy of the ground states is also observed. Thus, one may consider that the gapped state is the FQH state, which is confirmed by the numerical computation yielding *C*_tot_ = 1 [see Fig. 3(c)].

Therefore, we conclude that the FQH state emerges without the repulsive interaction (Re*V* = 0) which is adiabatically connected to the FQH state with *σ*_*xy*_ = 1/3 for the Hermitian case.

We stress that the bulk gap opens due to two-body loss inducing the effective repulsive interaction, which is reminiscent of the continuous quantum Zeno effect^66–68,77–81^. We note that in contrast to the case of Re*V* = 0, the second term may suppress the bulk gap for Re*V* > 0. Indeed, for *V* = *V*_*R*_ > 0 the prefactor of the second term of Eq. (10) has the opposite sign [−(Re*V*)^2^/*ħω*_0_]. Correspondingly, the second order term suppresses the bulk gap induced by the first term of Eq. (10), which can be numerically observed.

## Summary and Outlook

In this paper, by focusing on the FQH system at *ν* = 1/3, we have analyzed impacts of non-Hermiticity on topological ordered phases. We have elucidated the robustness of topological degeneracy against non-Hermitian interactions which arises from many-body translational symmetry. Combining the numerical results of the Chern number *C*_tot_ = 1 and the topological degeneracy leads us the conclusion that non-Hermitian Hamiltonian (1b) shows the FQH state. Furthermore, we have discovered that the FQH state emerges without repulsive interactions (*ReV* = 0). This intriguing behavior arises from the effective repulsion induced by the two-body loss, which is reminiscent of the continuous quantum Zeno effect.

We finish this article with comments on future directions. One of the significant issues is experimental realization of non-Hermitian fractional quantum Hall states. Although more detailed quantitative analysis is desired, we can make a rough estimation. Supposing that hopping is approximately ${t}_{0}/h \sim 1\,{\rm{kHz}}$^82^, the bulk gap (Δ) and the lifetime (*τ*) can be estimated as $\Delta /h \sim 10\,{\rm{Hz}}$ and $\tau  \sim 0.6\,{\rm{s}}$ for strong gauge field [see Fig. 2(a)]. Concerning the bulk gap, the energy scale is comparable with the temperature range, where the experiments are carried out^83^. However, the gap is enhanced by the strong repulsive interactions which can be tuned by the Feshbach resonance. Thus, we expect that experimental realization is accomplished for strong repulsive interactions. Furthermore, in Fig. 2(c), we have numerically observed that the Berry curvature is almost independent of *θ*’s, which implies that the computation of the Chern number may be simplified by defining the non-Hermitian counterpart of the one-plaquet Chern number for the Hermitian case^84–87^. We leave the extension of one-plaquet Chern number to non-Hermitian systems as a future work. In addition, we have observed that the interplay between the dissipative two-body interaction and the kinetic term yields four-body interactions which open the bulk gap and yield the FQH state. This unconventional mechanism of gap opening may provide new direction to access exotic topological ordered states induced by many-body interactions higher than two-body (e.g., the Moore-Read state^88,89^). Hunting such exotic topological ordered states is also left as a significant issue to be addressed.

## Supplementary Information

Supplementary

## References

[1] Thouless DJ, Kohmoto M, Nightingale MP, den Nijs M (1982). Quantized hall conductance in a two-dimensional periodic potential. Phys. Rev. Lett..

[2] Halperin BI (1982). Quantized hall conductance, current-carrying edge states, and the existence of extended states in a two-dimensional disordered potential. Phys. Rev. B.

[3] Hatsugai Y (1993). Chern number and edge states in the integer quantum hall effect. Phys. Rev. Lett..

[4] Kane CL, Mele EJ (2005). *Z*_2_ topological order and the quantum spin hall effect. Phys. Rev. Lett..

[5] Kane CL, Mele EJ (2005). Quantum spin hall effect in graphene. Phys. Rev. Lett..

[6] König M (2007). Quantum spin hall insulator state in hgte quantum wells. Science.

[7] Qi X-L, Hughes TL, Zhang S-C (2008). Topological field theory of time-reversal invariant insulators. Phys. Rev. B.

[8] Hasan MZ, Kane CL (2010). Colloquium. Rev. Mod. Phys..

[9] Qi X-L, Zhang S-C (2011). Topological insulators and superconductors. Rev. Mod. Phys..

[10] Pesin, D. & Balents, L. Mott physics and band topology in materials with strong spinju deng rbit interaction. *Nature Physics* **6**, 376 EP–, Article (2010).

[11] Manmana SR, Essin AM, Noack RM, Gurarie V (2012). Topological invariants and interacting one-dimensional fermionic systems. Phys. Rev. B.

[12] Yoshida T, Peters R, Fujimoto S, Kawakami N (2014). Characterization of a topological mott insulator in one dimension. Phys. Rev. Lett..

[13] Yoshida T, Kawakami N (2016). Topological edge mott insulating state in two dimensions at finite temperatures: Bulk and edge analysis. Phys. Rev. B.

[14] Wen X-G (1995). Topological orders and edge excitations in fractional quantum hall states. Advances in Physics.

[15] Tsui DC, Stormer HL, Gossard AC (1982). Two-dimensional magnetotransport in the extreme quantum limit. Phys. Rev. Lett..

[16] Laughlin RB (1983). Anomalous quantum hall effect: An incompressible quantum fluid with fractionally charged excitations. Phys. Rev. Lett..

[17] Jain JK (1989). Composite-fermion approach for the fractional quantum hall effect. Phys. Rev. Lett..

[18] Tang E, Mei J-W, Wen X-G (2011). High-temperature fractional quantum hall states. Phys. Rev. Lett..

[19] Sun K, Gu Z, Katsura H, Das Sarma S (2011). Nearly flatbands with nontrivial topology. Phys. Rev. Lett..

[20] Neupert T, Santos L, Chamon C, Mudry C (2011). Fractional quantum hall states at zero magnetic field. Phys. Rev. Lett..

[21] Sheng, D. N., Gu, Z.-C., Sun, K. & Sheng, L. Fractional quantum hall effect in the absence of landau levels. *Nature Communications* **2**, 389 EP–, Article (2011). 10.1038/ncomms1380 PMC3160145 21750543

[22] Regnault N, Bernevig BA (2011). Fractional chern insulator. Phys. Rev. X.

[23] Bergholtz EJ, Liu Z (2013). Topological flat band models and fractional chern insulators. International Journal of Modern Physics B.

[24] Kitaev A (2003). Fault-tolerant quantum computation by anyons. Annals of Physics.

[25] Kitaev Alexei (2006). Anyons in an exactly solved model and beyond. Annals of Physics.

[26] Hamma A, Zanardi P, Wen X-G (2005). String and membrane condensation on three-dimensional lattices. Phys. Rev. B.

[27] Takayama T (2015). Hyperhoneycomb iridate *β*–li_2_iro_3_ as a platform for kitaev magnetism. Phys. Rev. Lett..

[28] Kasahara Y (2018). Majorana quantization and half-integer thermal quantum hall effect in a kitaev spin liquid. Nature.

[29] Esaki K, Sato M, Hasebe K, Kohmoto M (2011). Edge states and topological phases in non-hermitian systems. Phys. Rev. B.

[30] Gong Z (2018). Topological phases of non-hermitian systems. Phys. Rev. X.

[31] Kawabata, K., Shiozaki, K., Ueda, M. & Sato, M. Symmetry and topology in non-hermitian physics. *arXiv preprint arXiv:1812*.*09133* (2018).

[32] Zhou H, Lee JY (2019). Periodic table for topological bands with non-hermitian symmetries. Phys. Rev. B.

[33] Katō, T. *Perturbation theory for linear operators*, vol. 132 (Springer, 1966).

[34] Shen, H., Zhen, B. & Fu, L. Topological band theory for non-hermitian hamiltonians. *arXiv preprint arXiv:1706*.*07435* (2017). 10.1103/PhysRevLett.120.146402 29694133

[35] Xu Y, Wang S-T, Duan L-M (2017). Weyl exceptional rings in a three-dimensional dissipative cold atomic gas. Phys. Rev. Lett..

[36] Kozii, V. & Fu, L. Non-hermitian topological theory of finite-lifetime quasiparticles: Prediction of bulk fermi arc due to exceptional point. *arXiv preprint arXiv:1708*.*05841* (2017).

[37] Yoshida T, Peters R, Kawakami N (2018). Non-hermitian perspective of the band structure in heavy-fermion systems. Phys. Rev. B.

[38] Carlström, J., Stålhammar, M., Budich, J. C. & Bergholtz, E. J. Knotted non-hermitian metals. *arXiv preprint arXiv:1810*.*12314* (2018).

[39] Ozcakmakli Turker Z, Yuce C (2019). Open and closed boundaries in non-hermitian topological systems. Phys. Rev. A.

[40] Budich JC, Carlström J, Kunst FK, Bergholtz EJ (2019). Symmetry-protected nodal phases in non-hermitian systems. Phys. Rev. B.

[41] Okugawa R, Yokoyama T (2019). Topological exceptional surfaces in non-hermitian systems with parity-time and parity-particle-hole symmetries. Phys. Rev. B.

[42] Yoshida T, Peters R, Kawakami N, Hatsugai Y (2019). Symmetry-protected exceptional rings in two-dimensional correlated systems with chiral symmetry. Phys. Rev. B.

[43] Zhou H, Lee JY, Liu S, Zhen B (2019). Exceptional surfaces in pt-symmetric non-hermitian photonic systems. Optica.

[44] Kawabata, K., Bessho, T. & Sato, M. Non-hermitian topology of exceptional points. *arXiv preprint arXiv:1902*.*08479* (2019).

[45] Yoshida, T. & Hatsugai, Y. Exceptional rings protected by emergent symmetry for mechanical systems. *arXiv preprint arXiv:1904*.*10764* (2019).

[46] Kimura, K., Yoshida, T. & Kawakami, N. Chiral-symmetry protected exceptional torus in correlated nodal-line semi-metals. *arXiv preprint arXiv:1905.11761, Phys. Rev. B*. **100**, 115124 (2019).

[47] Yao S, Wang Z (2018). Edge states and topological invariants of non-hermitian systems. Phys. Rev. Lett..

[48] Yao S, Song F, Wang Z (2018). Non-hermitian chern bands. Phys. Rev. Lett..

[49] Kunst FK, Edvardsson E, Budich JC, Bergholtz EJ (2018). Biorthogonal bulk-boundary correspondence in non-hermitian systems. Phys. Rev. Lett..

[50] Edvardsson E, Kunst FK, Bergholtz EJ (2019). Non-hermitian extensions of higher-order topological phases and their biorthogonal bulk-boundary correspondence. Phys. Rev. B.

[51] Lee CH, Thomale R (2019). Anatomy of skin modes and topology in non-hermitian systems. Phys. Rev. B.

[52] Borgnia, D. S., Kruchkov, A. J. & Slager, R.-J. Non-hermitian boundary modes. *arXiv preprint arXiv:1902*.*07217* (2019).

[53] Hatsugai Y, Ishibashi K, Morita Y (1999). Sum rule of hall conductance in a random quantum phase transition. Phys. Rev. Lett..

[54] Wilkin NK, Gunn JMF, Smith RA (1998). Do attractive bosons condense?. Phys. Rev. Lett..

[55] Schweikhard V, Coddington I, Engels P, Mogendorff VP, Cornell EA (2004). Rapidly rotating bose-einstein condensates in and near the lowest landau level. Phys. Rev. Lett..

[56] Ji A-C, Liu WM, Song JL, Zhou F (2008). Dynamical creation of fractionalized vortices and vortex lattices. Phys. Rev. Lett..

[57] Cooper N (2008). Rapidly rotating atomic gases. Advances in Physics.

[58] Furukawa S, Ueda M (2012). Quantum hall states in rapidly rotating two-component bose gases. Phys. Rev. A.

[59] Lin Y-J, Compton RL, Jiménez-García K, Porto JV, Spielman IB (2009). Synthetic magnetic fields for ultracold neutral atoms. Nature.

[60] Feshbach H (1958). Unified theory of nuclear reactions. Annals of Physics.

[61] Baumann K, Burdick NQ, Lu M, Lev BL (2014). Observation of low-field fano-feshbach resonances in ultracold gases of dysprosium. Phys. Rev. A.

[62] Scazza, F. *et al*. Observation of two-orbital spin-exchange interactions with ultracold su(n)-symmetric fermions. *Nature Physics* **10**, 779 EP–, Article (2014).

[63] Pagano G (2015). Strongly interacting gas of two-electron fermions at an orbital feshbach resonance. Phys. Rev. Lett..

[64] Höfer M (2015). Observation of an orbital interaction-induced feshbach resonance in ^173^Yb. Phys. Rev. Lett..

[65] Riegger L (2018). Localized magnetic moments with tunable spin exchange in a gas of ultracold fermions. Phys. Rev. Lett..

[66] Ashida Y, Furukawa S, Ueda M (2016). Quantum critical behavior influenced by measurement backaction in ultracold gases. Phys. Rev. A.

[67] Nakagawa M, Kawakami N, Ueda M (2018). Non-hermitian kondo effect in ultracold alkaline-earth atoms. Phys. Rev. Lett..

[68] Yamamoto, K. *et al*. Theory of non-hermitian fermionic superfluidity with a complex-valued interaction. *arXiv preprint arXiv:1903*.*04720* (2019). 10.1103/PhysRevLett.123.123601 31633989

[69] Ashida Y, Furukawa S, Ueda M (2017). Parity-time-symmetric quantum critical phenomena. Nature communications.

[70] Haldane FDM (1983). Fractional quantization of the hall effect: A hierarchy of incompressible quantum fluid states. Phys. Rev. Lett..

[71] Niu Q, Thouless DJ, Wu Y-S (1985). Quantized hall conductance as a topological invariant. Phys. Rev. B.

[72] Haldane FDM (1985). Many-particle translational symmetries of two-dimensional electrons at rational landau-level filling. Phys. Rev. Lett..

[73] Sheng DN (2003). Disorder-driven collapse of the mobility gap and transition to an insulator in the fractional quantum hall effect. Phys. Rev. Lett..

[74] Kudo K, Kariyado T, Hatsugai Y (2017). Many-body chern numbers of â€”Â» = 1/3 and 1/2 states on various lattices. Journal of the Physical Society of Japan.

[75] Fukui T, Hatsugai Y, Suzuki H (2005). Chern numbers in discretized brillouin zone: efficient method of computing (spin) hall conductances. Journal of the Physical Society of Japan.

[76] Fukui, T. & Hatsugai, Y. Quantum spin hall effect in three dimensional materials: Lattice computation of z2 topological invariants and its application to bi and sb. *Journal of the Physical Society of Japan* **76**, 053702–053702 (2007).

[77] Syassen N., Bauer D. M., Lettner M., Volz T., Dietze D., Garcia-Ripoll J. J., Cirac J. I., Rempe G., Durr S. (2008). Strong Dissipation Inhibits Losses and Induces Correlations in Cold Molecular Gases. Science.

[78] Mark MJ (2012). Preparation and spectroscopy of a metastable mott-insulator state with attractive interactions. Phys. Rev. Lett..

[79] Barontini G (2013). Controlling the dynamics of an open many-body quantum system with localized dissipation. Phys. Rev. Lett..

[80] Zhu B (2014). Suppressing the loss of ultracold molecules via the continuous quantum zeno effect. Phys. Rev. Lett..

[81] Tomita, T., Nakajima, S., Danshita, I., Takasu, Y. & Takahashi, Y. Observation of the mott insulator to superfluid crossover of a driven-dissipative bose-hubbard system. *Science Advances* **3**, https://advances.sciencemag.org/content/3/12/e1701513.full.pdf (2017). 10.1126/sciadv.1701513 PMC5744470 29291246

[82] Baier S (2016). Extended bose-hubbard models with ultracold magnetic atoms. Science.

[83] Mazurenko A (2017). A cold-atom fermi–hubbard antiferromagnet. Nature.

[84] Hastings MB, Michalakis S (2015). Quantization of hall conductance for interacting electrons on a torus. Communications in Mathematical Physics.

[85] Koma, T. Topological current in fractional chern insulators. *arXiv preprint arXiv:1504*.*01243* (2015).

[86] Watanabe H (2018). Insensitivity of bulk properties to the twisted boundary condition. Phys. Rev. B.

[87] Kudo K, Watanabe H, Kariyado T, Hatsugai Y (2019). Many-body chern number without integration. Phys. Rev. Lett..

[88] Moore G, Read N (1991). Nonabelions in the fractional quantum hall effect. Nuclear Physics B.

[89] Greiter M, Wen X-G, Wilczek F (1991). Paired hall state at half filling. Phys. Rev. Lett..

