# Supplementary material for: Non-Hermitian fractional quantum Hall states
Source: Sci Rep. 2019 Nov 15;9:16895. doi: 10.1038/s41598-019-53253-8 (PMC6858315; doi:10.1038/s41598-019-53253-8)
Supplement: Supplementary file 1 — Supplementary [file 41598_2019_53253_MOESM1_ESM.pdf]

# Supplementary of "Non-Hermitian fractional quantum Hall states"

Tsuneya Yoshida,[1] Koji Kudo,[1] and Yasuhiro Hatsugai[1]

[1] *Department of Physics, University of Tsukuba, Ibaraki 305-8571, Japan*

(Dated: August 27, 2019)

## I. DETAILED RESULTS OF THE HERMITIAN CASE

We here summarize the results of the Hermitian case[1–11]. It is well-known that the nearest neighbor interaction ($V > 0$) opens the bulk gap, separating exited states and the ground states whose topological degeneracy is three. Extrapolating the obtained bulk gap for each value of $N_f$, we can confirm that the bulk gap remains finite in the thermodynamic limit. We can also numerically confirm the three-fold degeneracy for the ground state multiplet[3]. The topological property of the gapped state can be characterized by many-body Chern number $C_{\mathrm{tot}}$ with twisting

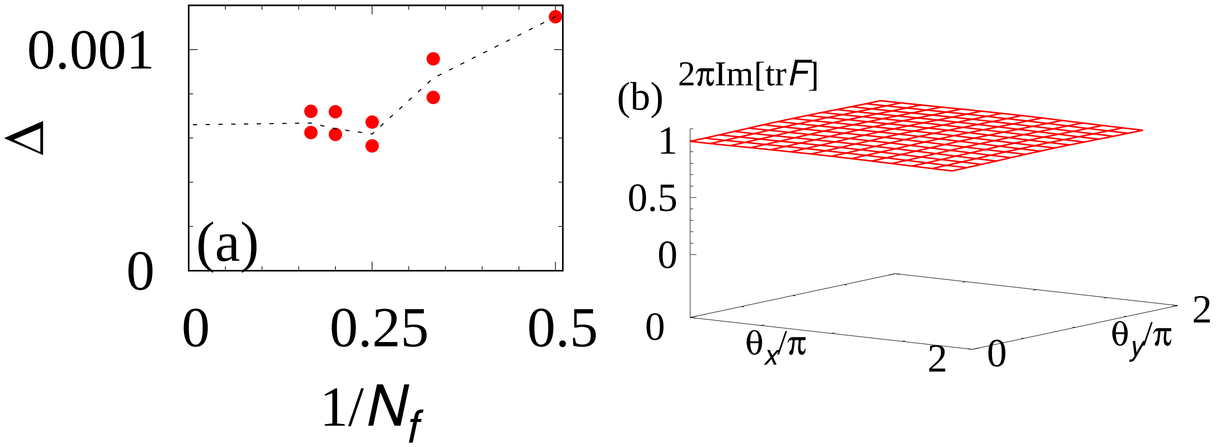


FIG. 1. (Color Online). (a) The bulk gap as a function of $N_f$ for $N_{\mathrm{keep}} = N_\phi$. These data are obtained in a similar way as Fig. 2(a) in the main text. (b) Berry curvature $\mathrm{tr}F/2\pi i$ as a function of $\theta_x$ and $\theta_y$.

the boundary condition. Computing the Chern number for the ground state multiplet yields $C_{\mathrm{tot}} = 1$[4].

The above numerical data indicate that the nearest neighbor interaction $V > 0$ resluts in the FQH state with $\sigma_{xy} = 1/3$.

## II. CHERN NUMBER AND BERRY CONNECTION

Here, we show that the Chern number defined in Eq. (5b) takes an integer.

Now, consider a two-dimensional parameter space $(\theta_x, \theta_y)$ with $0 \leq \theta_{x(y)} < 2\pi$. Then, we divide the two-dimensional space into two regions, I and II since taking the unique gauge may not be allowed. Because both of the gauges are available on the boundary of the region I and II, the eigenvectors are related to each other with an invertible matrix $M$;

$$|\Psi^{II}_n\rangle_R := |\Psi^{I}_{n'}\rangle_R M_{n'n}, \tag{1a}$$

$$_L\langle\Psi^{II}_n| := M^{-1}_{nn'}\,{}_L\langle\Psi^{I}_{n'}|, \tag{1b}$$

where the summation is taken over repeated indices.

Let us evaluate the integration of Eq. (5b). Applying Stokes' theorem, we can rewrite it as

$$C_{\mathrm{tot}} = \frac{1}{2\pi i}\int_C d\boldsymbol{\theta}\cdot\left(\mathrm{tr}\boldsymbol{A}^{I} - \mathrm{tr}\boldsymbol{A}^{II}\right), \tag{2a}$$

with

$$\boldsymbol{A}^{\alpha}_{nm} = {}_L\langle\Psi^{\alpha}_n|\boldsymbol{\nabla}\Psi^{\alpha}_m\rangle_R. \tag{2b}$$

Here, the integral of Eq. (2a) is taken along the boundary. $d\boldsymbol{\theta} := (d\theta_x, d\theta_y)$, and $\boldsymbol{\nabla} := (\partial/\partial\theta_x, \partial/\partial\theta_y)$.

Eq. (2a) can be further simplified as follows:

$$\begin{aligned} C_{\rm tot} &= \frac{1}{2\pi i}\int_C d\boldsymbol{\theta}\cdot {\rm tr}\left(M^{-1}\boldsymbol{\nabla} M\right) \\ &= \frac{1}{2\pi i}\int_C d\boldsymbol{\theta}\cdot \boldsymbol{\nabla}{\rm tr}\log M \\ &= \frac{1}{2\pi i}\int_C d\boldsymbol{\theta}\cdot \boldsymbol{\nabla}\log {\rm det} M. \end{aligned} \tag{3}$$

Noticing that det$M$ is a single-valued function, we can see that the integral is reduced to the winding number

$$\begin{aligned} C_{\rm tot} &= \frac{1}{2\pi}{\rm Im}\int_C d\boldsymbol{\theta}\cdot \boldsymbol{\nabla}\log {\rm det} M, \\ &\in \mathbb{Z}. \end{aligned} \tag{4}$$

Therefore, we can conclude that the Chern number takes integer.

## III. DERIVATION OF EQ. (7)

As mentioned in the main text, Eq. (7) can be obtained for the Landau gauge with $N_x = N_y$.

As a preparation, we discuss the translational symmetry in term of the eigenvectors (4b). The state $|\varphi_\alpha(k_y)\rangle$ can be expanded as

$$|\varphi_\alpha(k_y)\rangle = \sum_{i_x,i_y} \varphi_{i_x i_y \alpha} c^\dagger_{i_x i_y}|0\rangle. \tag{5}$$

Because $|\varphi_\alpha(k_y)\rangle$ is an eigenstate of $T_y$ [see Eq. (6)], we have

$$\varphi_{i_x i_y -1 \alpha} = e^{-ik_y}\varphi_{i_x i_y \alpha}. \tag{6}$$

Now we show that Eq. (7) holds. This can be seen by analysing whether $U_G|\varphi_\alpha(k_y)\rangle$ is an eigenstate of $T_y$;

$$\begin{aligned} T_y U_G|\varphi_\alpha(k_y)\rangle &= \sum_{j_x=1}^{N_x}\sum_{j_y=1}^{N_y} e^{-i2\pi\phi j_y} T_y \varphi_{j_x j_y \alpha} c^\dagger_{j_x j_y}|0\rangle \\ &= \sum_{j_x=1}^{N_x}\sum_{j_y=1}^{N_y} e^{-i2\pi\phi j_y} \varphi_{j_x j_y \alpha} c^\dagger_{j_x j_y+1}|0\rangle \\ &= \sum_{j_x=1}^{N_x}\sum_{j_y=2}^{N_y+1} e^{-i2\pi\phi (j_y-1)} \varphi_{j_x (j_y-1) \alpha} c^\dagger_{j_x j_y}|0\rangle \\ &= \sum_{j_x=1}^{N_x}\sum_{j_y=2}^{N_y+1} e^{-i2\pi\phi (j_y-1)-ik_y} \varphi_{j_x j_y \alpha} c^\dagger_{j_x j_y}|0\rangle \\ &= \sum_{j_x=1}^{N_x} e^{-ik_y}\left[\sum_{j_y=1}^{N_y} e^{-i2\pi\phi (j_y-1)} \varphi_{j_x j_y \alpha} c^\dagger_{j_x j_y}|0\rangle + \left(e^{-i2\pi\phi N_y}-1\right)\varphi_{j_x 1\alpha} c^\dagger_{j_x 1}|0\rangle\right] \\ &= e^{-i(k_y-2\pi\phi)} U_G|\varphi_\alpha(k_y)\rangle. \end{aligned} \tag{7}$$

Here, from the third to the fourth line we have used Eq. (6). From fifth to the last line, we have used the relation $\phi N_y = 1$ which is satisfied for the system with the Landau gauge and for $N_x = N_y$.

[1] R. B. Laughlin, Phys. Rev. Lett. **50**, 1395 (1983).
[2] Q. Niu, D. J. Thouless, and Y.-S. Wu, Phys. Rev. B **31**, 3372 (1985).

[3] F. D. M. Haldane, Phys. Rev. Lett. **55**, 2095 (1985).
[4] D. N. Sheng, X. Wan, E. H. Rezayi, K. Yang, R. N. Bhatt, and F. D. M. Haldane, Phys. Rev. Lett. **90**, 256802 (2003).
[5] E. Tang, J.-W. Mei, and X.-G. Wen, Phys. Rev. Lett. **106**, 236802 (2011).
[6] K. Sun, Z. Gu, H. Katsura, and S. Das Sarma, Phys. Rev. Lett. **106**, 236803 (2011).
[7] T. Neupert, L. Santos, C. Chamon, and C. Mudry, Phys. Rev. Lett. **106**, 236804 (2011).
[8] D. N. Sheng, Z.-C. Gu, K. Sun, and L. Sheng, Nature Communications **2**, 389 EP (2011), article.
[9] N. Regnault and B. A. Bernevig, Phys. Rev. X **1**, 021014 (2011).
[10] E. J. Bergholtz and Z. Liu, International Journal of Modern Physics B **27**, 1330017 (2013), https://doi.org/10.1142/S021797921330017X.
[11] K. Kudo, T. Kariyado, and Y. Hatsugai, Journal of the Physical Society of Japan **86**, 103701 (2017), https://doi.org/10.7566/JPSJ.86.103701.
